# Supplementary material for: Differential modulation of positive and negative prediction errors by stimulus variability in the mouse posterior parietal cortex
Source: Commun Biol. 2025 Sep 30;8:1397. doi: 10.1038/s42003-025-08797-z (PMC12484734; doi:10.1038/s42003-025-08797-z)
Supplement: Supplementary file 2 — Supplementary Information [file 42003_2025_8797_MOESM2_ESM.pdf]

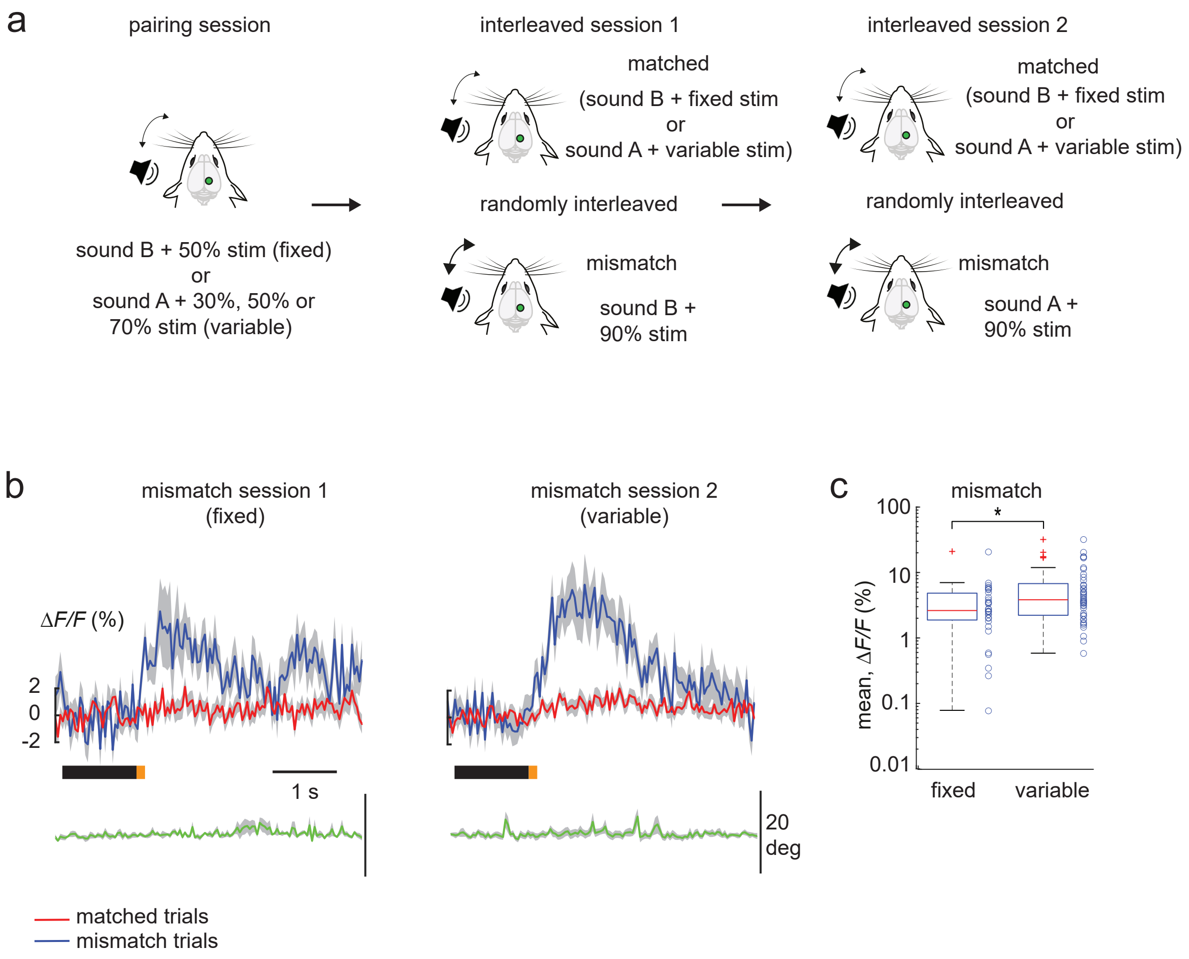

Supplementary Fig.1 Positive prediction errors increase with whisker stimulus variability is not influenced by the type of sound.
a Experimental design to evaluate positive prediction errors under stimulus variability in a single imaging session. During the pairing session, sound B is paired with a fixed whisker stimulus intensity of 50%, and sound A with a variable whisker stimulus intensity (30%, 50%, or 70%). Interleaved session 1 and 2 presented positive mismatch trials where the whisker stimulus intensity was randomly increased to 90% in 15% of trials. Hence, the interleaved sessions comprised of randomized matched (sound B with 50% whisker stimulus intensity, sound A with a variable whisker stimulus intensity of 30%, 50% or 70%) and mismatch trials (session 1: sound B with 90% whisker intensity; session 2: sound A with 90% whisker intensity). **b** Population averages of ΔF/F traces of mismatch-responsive neurons in session 1 (fixed whisker stimulus intensity, n = 31 mismatch neurons of 431 neurons) and session 2 (variable whisker stimulus intensity, n= 41 mismatch neurons of 431 neurons), with their corresponding matched (red) and mismatch (blue) trial averages (4 Thy-1GCaMP6f mice; 7 FOV). **c** Box plot of average population responses of mismatch-responsive neurons, for interleaved sessions 1 (fixed whisker stimulus intensity) and 2 (variable whisker stimulus intensity) as shown in **b**. mean response ΔF/F: fixed whisker stimulus intensity 3.6 ± 0.7%, variable whisker stimulus intensity 6.3 ± 1.0%. Boxplot central line indicates the median, the bottom and top edges of the box indicate the 25th and 75th percentiles respectively, the whiskers extend to maximum and minimum points within 1.5 s.d., and outliers are marked with crosses. Data are represented as mean ± s.e.m. Statistical significance is indicated by * for $p < 0.05$ with two-sided Wilcoxon-Mann-Whitney test. Mouse illustration in **a** is adapted from [6].

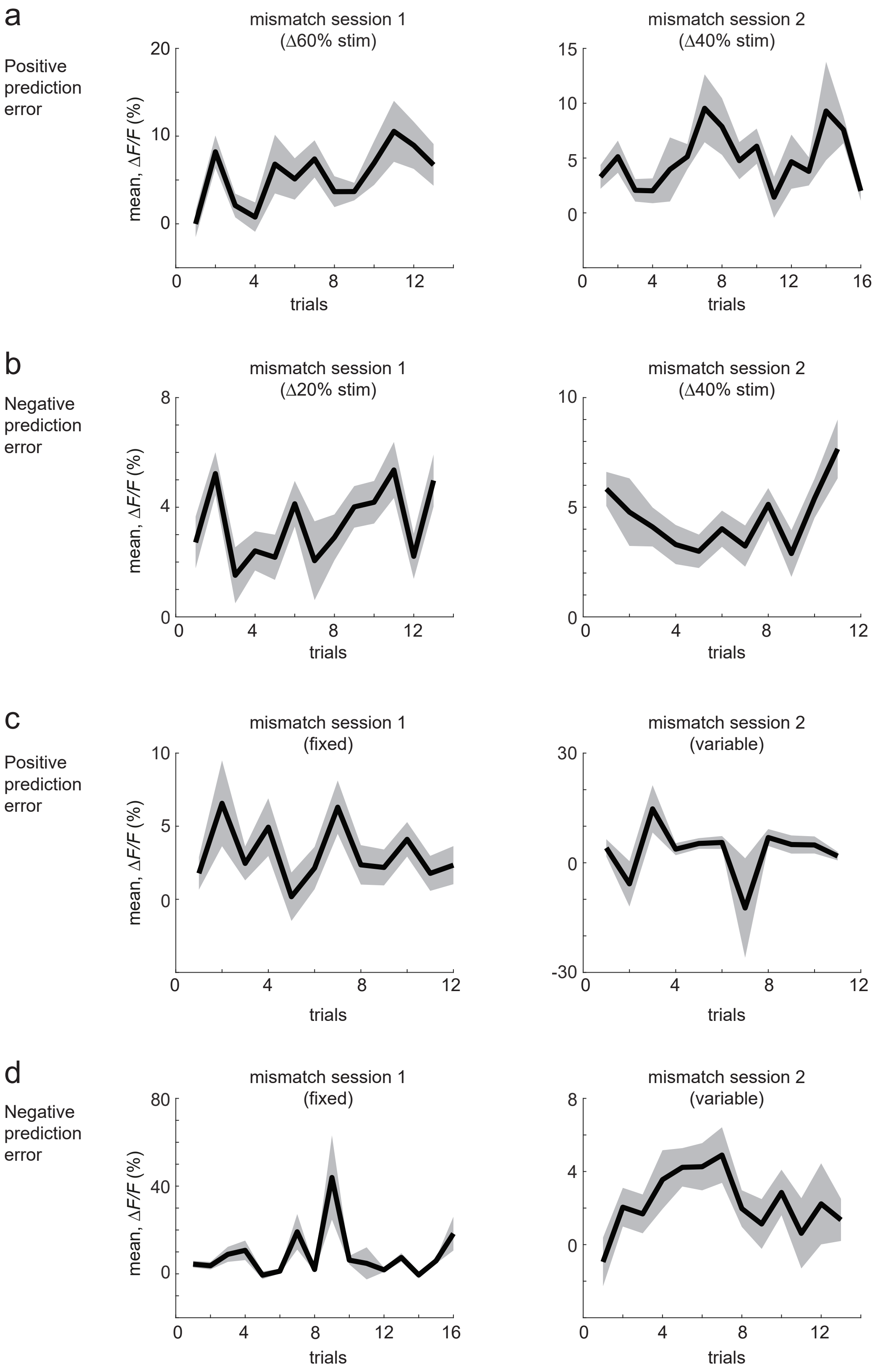

Supplementary Fig.2 Mismatch responses remain stable across mismatch trials.
a-**d** Average population responses of mismatch-responsive neurons across successive mismatch trials for positive prediction errors (**a**, **c**) and negative prediction errors (**b**, **d**) in the fixed (**a**, **b**) and variable (**c**, **d**) conditions. The number of trials shown is determined by the session with the least number of mismatch trials. Data are presented as mean ± s.e.m.

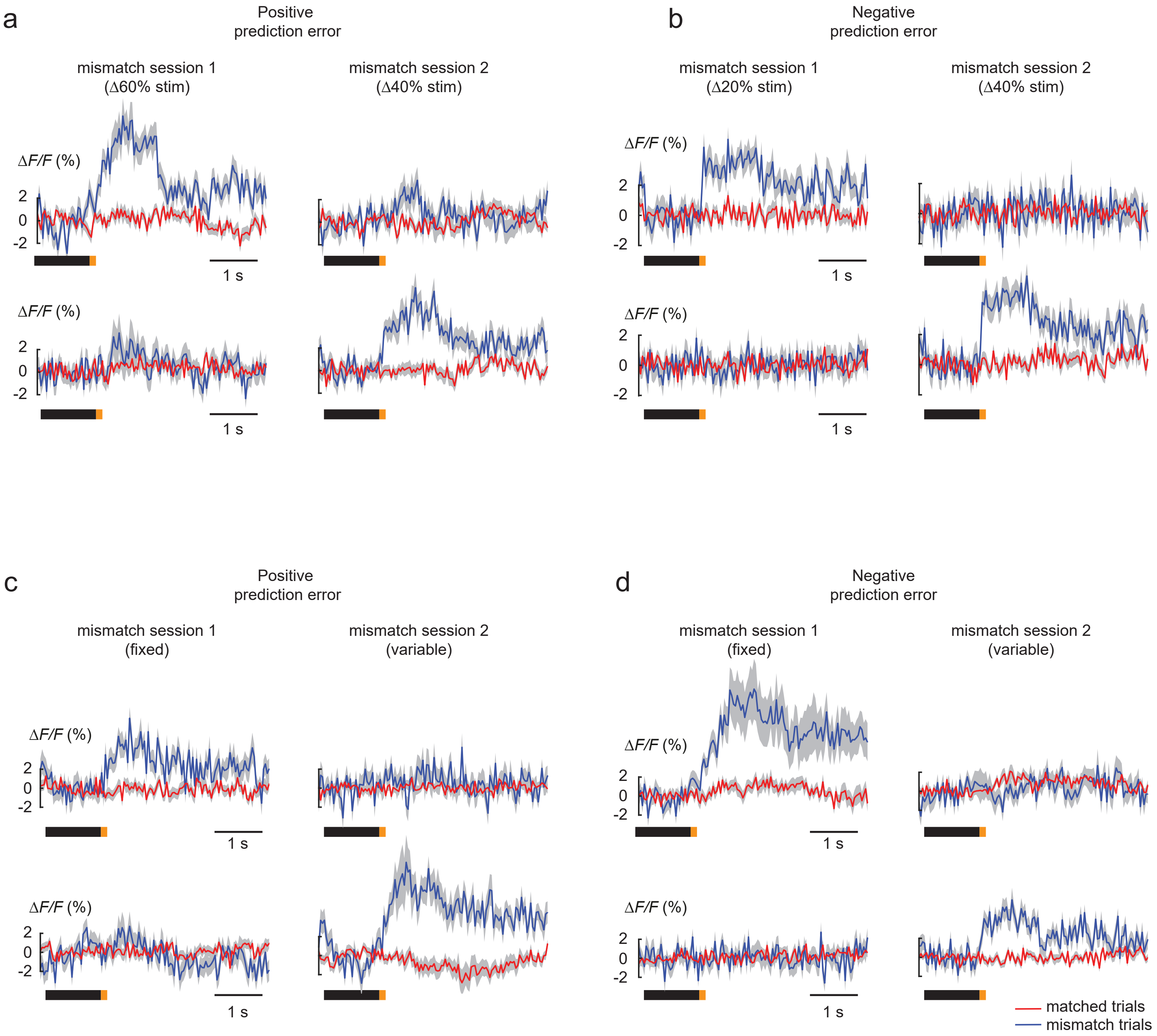

Supplementary Fig.3 Mismatch neurons are largely responsive in their respective mismatch sessions.
a Population averages of ΔF/F traces of positive mismatch-responsive neurons from Fig.1 in session 1 and their respective response in session 2, along with the population averages of mismatch responsive neurons in session 2 and their respective response in session 1. **b-d**, same as a for the mismatch responsive neurons from Fig.2 to 4 respectively. Population traces correspond to the matched (red) and mismatch (blue) trial averages. Data are presented as mean ± s.e.m.

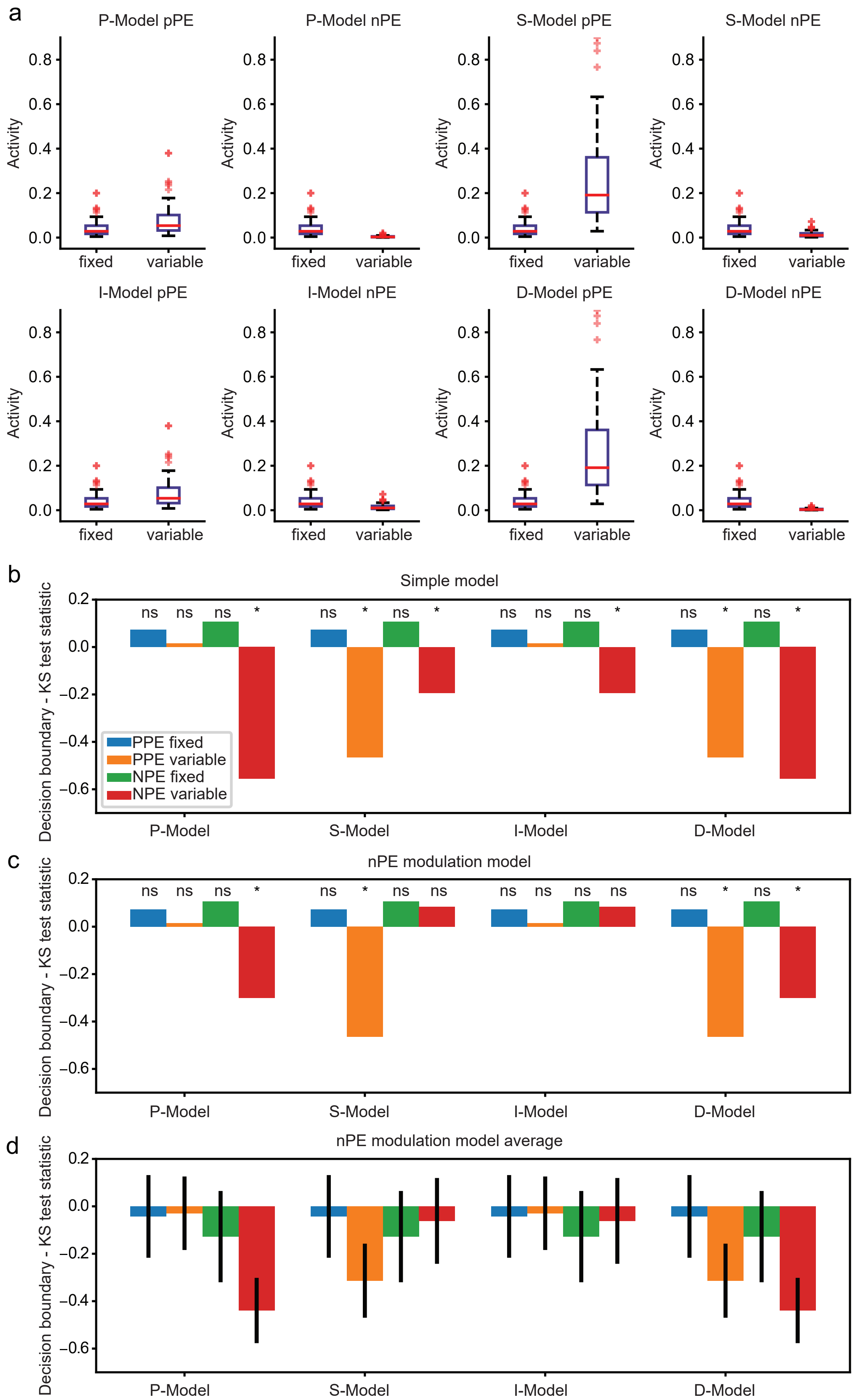

Supplementary Fig.4 Further modelling results and Kolmogorov-Smirnovtest statistics for comparing models with the data.

a Activity of nPE and pPE neurons in the different models as in Fig. 5b-e for the simple case without nPE modulation. b-c Difference of the Two-sample Kolmogorov-Smirnov test statistic to the decision boundary for each modelled distribution for the simple case **(b)** and the case with additional nPE modulation by variability **(c)**. The null hypothesis that the samples come from the same underlying distribution is rejected at level alpha=0.05 if the test statistic is larger than the decision boundary. Hence, positive values indicate that the null hypothesis cannot be rejected, whereas negative values indicate that it is rejected. The star marks p-values < 0.05. The decision boundary is defined as $c(\alpha)\sqrt{(n+m)/(n\cdot m)}$, where $c(\alpha)=\sqrt{-\ln(\alpha/2)\cdot 1/2}$, and n and m are the sample sizes. **d** Difference of KS test statistic to the decision boundary averaged over 20 different seeds. Boxplot central line indicates the median, the bottom and top edges of the box indicate the 25th and 75th percentiles respectively, the whiskers extend to maximum and minimum points within 1.5 s.d., and outliers are marked with crosses. Error bars denote standard deviation.

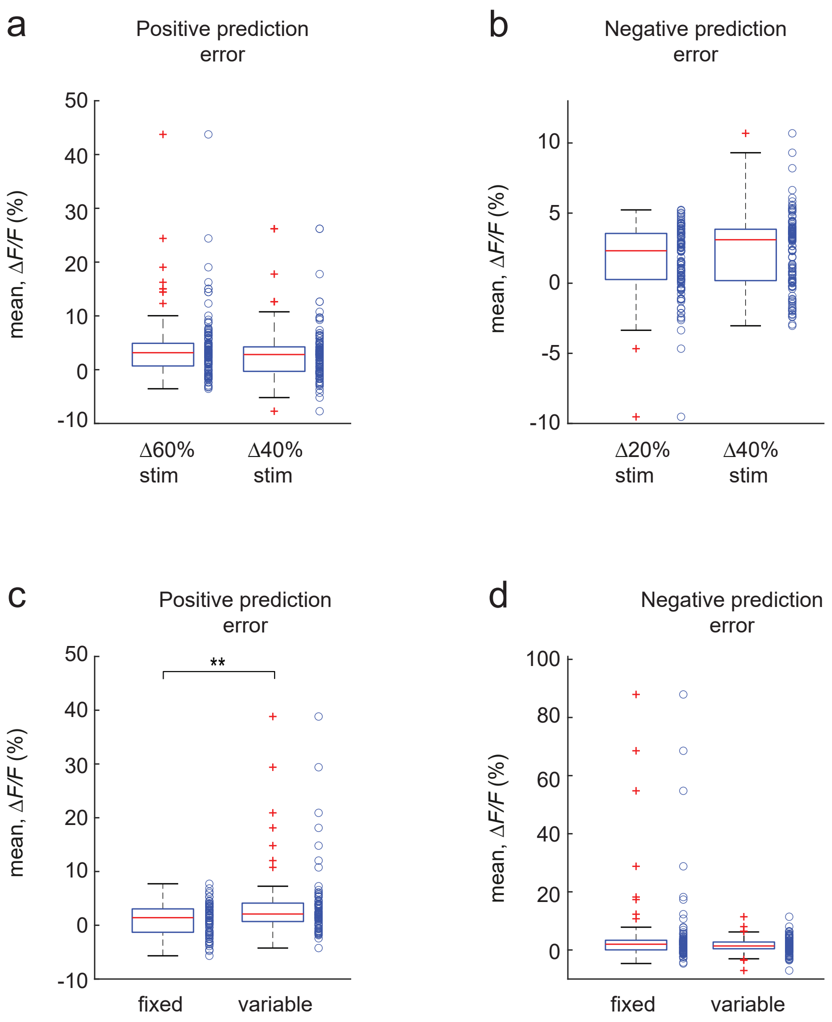

Supplementary Fig.5 Prediction error neurons respond mainly to one mismatch session

a Box plot of average population responses of all mismatch-responsive neurons (pooled across inter-leaved session 1 and 2) for Experiment 1. Mean response ΔF/F: Δ60% whisker stimulus intensity 3.9 ± 0.5%, Δ40% whisker stimulus intensity 2.9 ± 0.4%, n = 119 mismatch neurons. **b** same as **a**, but for Experiment 2. Mean response ΔF/F: Δ20% whisker stimulus intensity 1.7 ± 0.2%, Δ40% whisker stimulus intensity 2.3 ± 0.2%, n = 116 mismatch neurons. **c** same as **a**, but for Experiment 3. Mean response ΔF/F: fixed whisker stimulus intensity 1.1 ± 0.3%, variable whisker stimulus intensity 3.7 ± 0.7%, n = 77 mismatch neurons. **d** same as **a**, but for Experiment 4. Mean response ΔF/F: fixed whisker stimulus intensity 4.6 ± 1.3%, variable whisker stimulus intensity 1.6 ± 0.3%, n = 93 mismatch neurons. Boxplot central line indicates the median, the bottom and top edges of the box indicate the 25th and 75th percentiles respectively, the whiskers extend to maximum and minimum points within 1.5 s.d., and outliers are marked with crosses. Statistical significance is indicated by ** for $p < 0.01$ with two-sided Wilcoxon signed-rank test for paired data.

Supplementary Table 1.

Experiment 1

mouse	FOV	interleaved 1	interleaved 2	overlap	Total cells	Total fraction
m186	1	15	6	0	65	32.3
m188	1	16	9	2	80	28.9
m234	1	5	15	0	123	16.2
	2	11	5	2	118	11.9
m235	1	3	7	1	91	9.9
	2	5	5	0	73	13.7
m239	1	3	3	0	35	17.1
	2	5	6	0	118	9.3

Experiment 2

mouse	FOV	interleaved 1	interleaved 2	overlap	Total cells	Total fraction
m188	1	15	12	0	128	21.1
m234	1	7	12	0	83	22.9
m235	1	13	14	0	108	25.0
	2	5	8	1	92	13.0
m239	1	2	3	0	33	15.2
	2	3	7	0	54	18.5
	3	10	5	0	79	19.0

Experiment 3

mouse	FOV	interleaved 1	interleaved 2	overlap	Total cells	Total fraction
m188	1	13	8	0	85	24.7
m234	1	1	11	0	73	16.4
	2	4	10	0	67	20.9
m235	1	6	2	0	41	19.5
m239	1	8	4	0	56	21.4
	2	3	7	0	54	18.5

Experiment 4

mouse	FOV	interleaved 1	interleaved 2	overlap	Total cells	Total fraction
m186	1	2	2	0	35	11.4
m188	1	13	6	0	90	21.1
m234	1	15	16	0	143	21.7
m239	1	4	3	0	40	17.5
	2	10	11	0	104	20.2
	3	7	4	0	66	11.1

Summary of FOVs acquired in Experiment 1 to 4.

A table indicating the number of mismatch neurons identified per interleaved session, along with their overlap between the sessions and the total number of neurons for that particular FOV. For each experiment (Figures 1 to 4) the data is separated at the mouse level and FOV level.

Supplementary Table 2.

Experiment 1	Model	SE	p-value	95%CI	n (cells)	n (mice)
MouseID as random effect	Standard LME	0.009	0.048	[-0.038, -0.0002]	119	5
	GLME (Gamma)	0.131	0.113	[-0.469, 0.050]	119	5
	Log-transformed LME	0.092	0.064	[-0.354, 0.010]	119	5
	Permutation Test		0.0496		119	5

Experiment 2	Model	SE	p-value	95%CI	n (cells)	n (mice)
MouseID as random effect	Standard LME	0.002	0.002	[0.003, 0.012]	116	4
	GLME (Gamma)	0.055	0.0004	[0.090, 0.308]	116	4
	Log-transformed LME	0.056	0.0005	[0.089, 0.311]	116	4
	Permutation Test		0.0013		116	4

Experiment 3	Model	SE	p-value	95%CI	n (cells)	n (mice)
MouseID as random effect	Standard LME	0.013	0.025	[0.004, 0.054]	77	4
	GLME (Gamma)	0.227	0.004	[0.219, 1.122]	77	4
	Log-transformed LME	0.177	0.007	[0.137, 0.841]	77	4
	Permutation Test		0.014		77	4

Experiment 4	Model	SE	p-value	95%CI	n (cells)	n (mice)
MouseID as random effect	Standard LME	0.024	0.065	[–0.091, 0.003]	93	4
	GLME (Gamma)	0.169	0.016	[–0.752, –0.079]	93	4
	Log-transformed LME	0.173	0.036	[–0.712, –0.024]	93	4
	Permutation Test		0.059		93	4

Summary of linear mixed-effects model (LME) analysis for Experiments 1 to 4.

The table reports results from four models for the four experiments: Standard LME, Generalized LME (Gamma distribution), Log-transformed LME, and a non-parametric permutation test. Analysis was performed using Mouse ID as the random effect (unfixed), with interleaved session 1 vs 2 as the fixed effect. For each model, the standard error (SE) is shown, along with the p-value, and 95% confidence interval. Cell counts and the number of random effect levels (mice) are also reported. Permutation test p-values are based on 10,000 label shuffles of the fixed effect.

Supplementary Table 3.

Experiment 1	Model	SE	p-value	95%CI	n (cells)	n (mice)
MouseID as random effect	Standard LME	0.0067301	0.1425	[-0.0232, 0.0034]	238	5
	GLME (Gamma)	0.1183	0.3604	[-0.3420, 0.1250]	178	5
	Log-transformed LME	0.1219	0.3926	[-0.3449, 0.1360]	178	5
	Permutation Test		0.1492		238	5

Experiment 2	Model	SE	p-value	95%CI	n (cells)	n (mice)
MouseID as random effect	Standard LME	0.00328	0.0899	[-0.00088, 0.01204]	232	4
	GLME (Gamma)	0.0851	0.0086	[0.0581, 0.3940]	184	4
	Log-transformed LME	0.14809	0.29666	[-0.1372, 0.44719]	184	4
	Permutation Test		0.0907		232	4

Experiment 3	Model	SE	p-value	95%CI	n (cells)	n (mice)
MouseID as random effect	Standard LME	0.00810	0.00155	[0.0101, 0.0421]	154	4
	GLME (Gamma)	0.18441	0.0161	[0.0853, 0.8160]	115	4
	Log-transformed LME	0.21859	0.1125	[–0.0835, 0.7827]	115	4
	Permutation Test		0.0006		154	4

Experiment 4	Model	SE	p-value	95%CI	n (cells)	n (mice)
MouseID as random effect	Standard LME	0.01317	0.0202	[–0.0568, –0.0049]	186	4
	GLME (Gamma)	0.16361	0.00182	[–0.8435, –0.1966]	142	4
	Log-transformed LME	0.17075	0.0661	[–0.6539, 0.0213]	142	4
	Permutation Test		0.0072		186	4

Summary of linear mixed-effects model (LME) analysis using all mismatch responsive neurons (pooled from interleaved session 1 and 2) for Experiments 1 to 4.

The table reports results from four models for the four experiments: Standard LME, Generalized LME (Gamma distribution), Log-transformed LME, and a non-parametric permutation test. Analysis was performed using Mouse ID as the random effect (unfixed), with interleaved session 1 vs 2 as the fixed effect. For each model, the standard error (SE) is shown, along with the p-value, and 95% confidence interval. Cell counts and the number of random effect levels (mice) are also reported. Permutation test p-values are based on 10,000 label shuffles of the fixed effect.
