# Supplementary material for: Differential modulation of positive and negative prediction errors by stimulus variability in the mouse posterior parietal cortex
Source: Commun Biol. 2025 Sep 30;8:1397. doi: 10.1038/s42003-025-08797-z (PMC12484734; doi:10.1038/s42003-025-08797-z)
Supplement: Supplementary file 3 — Description of Additional Supplementary Files [file 42003_2025_8797_MOESM3_ESM.pdf]

Description of Additional Supplementary Files

File name: Supplementary Data

Description: Source data
